# Respiratory Syncytial Virus Disease Is Mediated by Age-Variable IL-33

**DOI:** 10.1371/journal.ppat.1005217

**Published:** 2015-10-16

**Authors:** Jordy Saravia, Dahui You, Bishwas Shrestha, Sridhar Jaligama, David Siefker, Greg I. Lee, Jeffrey N. Harding, Tamekia L. Jones, Cynthia Rovnaghi, Bindiya Bagga, John P. DeVincenzo, Stephania A. Cormier

**Affiliations:** 1 Department of Pediatrics, University of Tennessee Health Science Center, Memphis, Tennessee, United States of America; 2 Children’s Foundation Research Institute at Le Bonheur Children’s Hospital, Memphis, Tennessee, United States of America; 3 Department of Microbiology, Immunology, and Biochemistry, University of Tennessee Health Science Center, Memphis, Tennessee, United States of America; St. Jude Children’s Research Hospital, UNITED STATES

## Abstract

Respiratory syncytial virus (RSV) is the most common cause of infant hospitalizations and severe RSV infections are a significant risk factor for childhood asthma. The pathogenic mechanisms responsible for RSV induced immunopathophysiology remain elusive. Using an age-appropriate mouse model of RSV, we show that IL-33 plays a critical role in the immunopathogenesis of severe RSV, which is associated with higher group 2 innate lymphoid cells (ILC2s) specifically in neonates. Infection with RSV induced rapid IL-33 expression and an increase in ILC2 numbers in the lungs of neonatal mice; this was not observed in adult mice. Blocking IL-33 with antibodies or using an IL-33 receptor knockout mouse during infection was sufficient to inhibit RSV immunopathogenesis (i.e., airway hyperresponsiveness, Th2 inflammation, eosinophilia, and mucus hyperproduction); whereas administration of IL-33 to adult mice during RSV infection was sufficient to induce RSV disease. Additionally, elevated IL-33 and IL-13 were observed in nasal aspirates from infants hospitalized with RSV; these cytokines declined during convalescence. In summary, IL-33 is necessary, either directly or indirectly, to induce ILC2s and the Th2 biased immunopathophysiology observed following neonatal RSV infection. This study provides a mechanism involving IL-33 and ILC2s in RSV mediated human asthma.

## Author Summary

IL-33 is responsible for the immunopathophysiological response observed following neonatal RSV infection in mice. Its presence in nasal aspirates of human infants with severe RSV and suggests its role in disease severity and asthma.

## Introduction

Respiratory syncytial virus (RSV) is the most common cause of lower respiratory tract infections in infants [1, 2], and is globally responsible for an estimated 64 million cases and 160,000 deaths each year [2]. In infants, severe RSV infection is characterized by bronchiolitis, interstitial pneumonitis, alveolitis [3], and a T helper 2 (Th2)-biased immune response in the lungs (i.e., Th2 cells, eosinophilia, mucus). One important correlate of severe RSV infection is age; most severe disease occurs in children <1 yr of age [4], with highest hospitalization rates occurring in those <6 months of age [5]. More recently, our understanding of RSV has been aided by the use of an age-appropriate mouse model in which neonatal mice infected with RSV exhibit an immunological (Th2 biased) and pathophysiological (pulmonary inflammation, eosinophilia, mucus hyperproduction, and long-term airways dysfunction) phenotype typically seen in human infants with severe RSV [6, 7].

We previously observed an early increase in IL-13 in the lungs of neonatal, but not adult mice, infected with RSV, which could not be explained by Th2 cells. Group 2 innate lymphoid cells (ILC2s) are a recently identified cell population naturally resident to the lungs that rapidly respond to IL-33 via its receptor ST2 to produce high levels of IL-13 [8]. It has been demonstrated that ILC2s play a critical role in the induction of Th2 immune responses [8–10]. Though ILC2s and IL-33 have both been closely associated with Th2 immunity, there are no data discerning either’s roles in the initiation and/or perpetuation of Th2 responses observed in infant RSV infection. This comes despite the fact that numerous studies show high correlation between genetic variation in the *IL33* or *ST2* genes and risk for asthma or severe RSV disease [11–13]–including a recent meta-analysis of GWAS studies which identifies *ST2* as one of the top ten loci that influence allergic sensitization [14]. This information, combined with the fact that ILC2s appear to be essential for early production of IL-13 during viral infections [15], makes ILC2s and IL-33 prime targets for the study of Th2-biased infant RSV disease.

In the present study, we show that IL-33 is rapidly secreted in the lungs of neonatal mice infected with RSV, which is accompanied by an increase in lung ILC2s. This response is age-specific, as RSV infection in adult mice does not induce increases in IL-33 or ILC2s. We further demonstrate that Th2-biased immunopathophysiology that occurs upon reinfection with RSV is IL-33-dependent.

## Results

### RSV induces robust, rapid IL-33 and IL-13 production in the lungs of neonates

**Fig 1:**
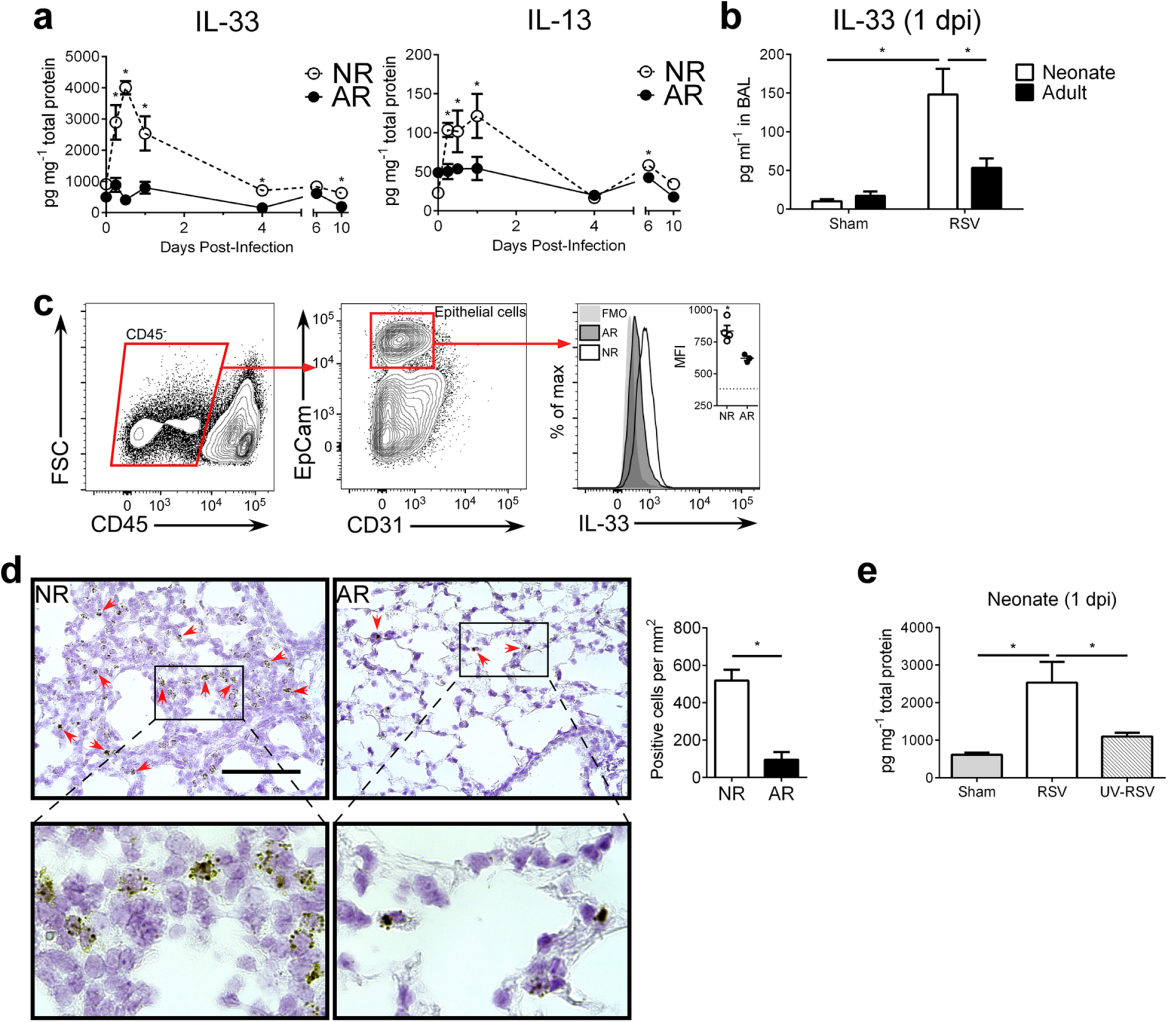
RSV induces robust, rapid IL-33 and IL-13 production in the lungs of neonates. (**a**) Cytokine protein levels of IL-33 and IL-13 in whole lung homogenates of RSV-infected neonate (5-day-old; NR) and adult (6-8-week-old; AR) mice (*n* = 4–10 per group) at different days (0–10) post-infection (dpi). (**b**) Cytokine protein levels of IL-33 detected in BAL at 1 dpi from NR and AR mice (*n* = 8–10 per group) compared to controls. (**c**) Gating strategy for determination of IL-33 expression by median fluorescence intensity (MFI) in epithelial cells (CD45^-^ EpCam^+^) with representative IL-33 MFI histogram (quantified in inset vs. fluorescence-minus-one (FMO) control (dotted line)). (**d**) Representative micrographs of *in situ* hybridization for IL-33 mRNA with red arrows to indicate positive staining cells (top), magnified inset (bottom), and quantification of IL-33 mRNA positive cells per unit area of lung. Scale bar = 100 μm. (**e**) Cytokine protein levels of IL-33 in whole lung homogenates of neonates infected with RSV or UV-RSV compared to control at 1 dpi. *P < 0.05 (Student’s *t*-test; **a**, **c**, **d**) (Two-way ANOVA; **b**) (One-way ANOVA; **e**). Data are representative of two (**a**, **c**) or 2 pooled (**b**, **d**, **e**) independent experiments (means ± s.e.m).

We previously observed an increase in pulmonary IL-13 immediately following RSV infection of neonatal, but not, adult mice. Since this increase was prior to the induction of Th2 cells and responses, it suggested a role for ILC2s. To determine if there are age-dependent differences in the induction of ILC2s in response to RSV, we infected neonatal mice (5 d of age; NR) and adult mice (6–8 weeks of age; AR) then kinetically measured IL-33 and IL-13 cytokine levels in whole lung homogenates (Fig 1a). Highly elevated IL-33 levels were detected in neonates as early as 0.25 days post-infection (dpi) and peaking at 0.5 dpi. Although IL-33 could be detected in the lungs of adult mice, there was no significant change in expression following RSV infection. A similar trend was observed with IL-13 levels—increased expression in the lungs of mice infected as neonates, which peaked at 1 dpi, and no significant change in the lungs of mice infected as adults. An additional significant increase in IL-13 was observed at 6 dpi in mice infected as neonates. Significantly elevated levels of IL-33 were also observed in bronchoalveolar lavage (BAL) from infected neonates but not adults (Fig 1b). Investigation into the cellular sources of IL-33 at 1 dpi revealed significant expression in airway epithelial cells (CD45^-^ EpCam^+^) of infected neonates (Fig 1c). A similar trend was observed with detection of IL-33 mRNA by *in situ* hybridization (Fig 1d). To determine if this rapid IL-33 increase in neonates requires live replicating virus, we “infected” neonates with UV-inactivated RSV (UV-RSV) and measured IL-33 in lung homogenates at 1 dpi (Fig 1e). IL-33 levels in the lungs following administration of UV-RSV was statistically no different than sham infected neonatal control mice and significantly lower than RSV infected neonatal mice.

### The percentage of ILC2s in the lungs of neonatal mice are further increased after RSV infection

**Fig 2:**
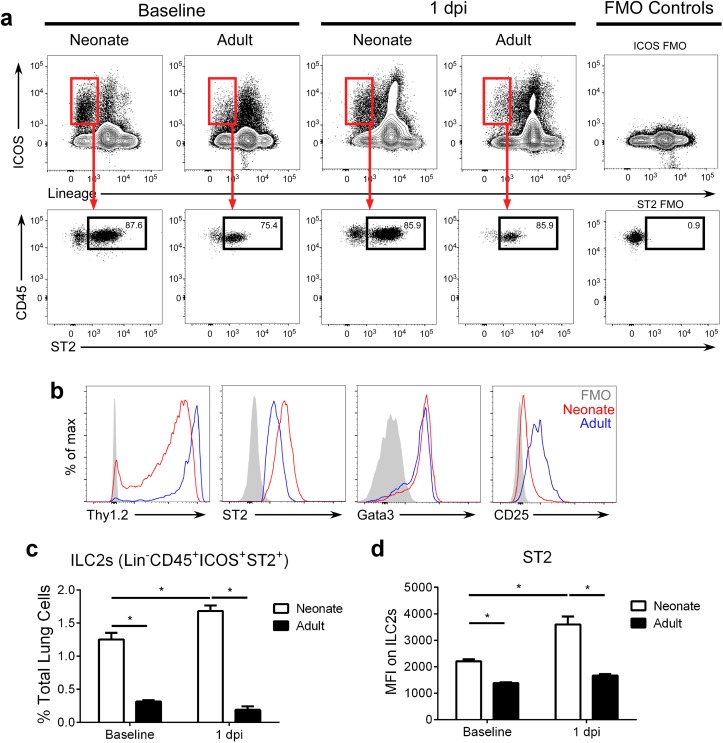
The percentage of ILC2s in the lungs of neonatal mice are further increased after RSV infection. (**a**) Gating strategy and (**b**) ILC2 marker expression on ILC2s at baseline and 1 dpi. (**c**) ILC2s as percent of total lung cells, and (**d**) MFI of ST2 expression of/on ILC2s at baseline and 1 dpi. ILC2s defined as lineage^-^ CD45^+^ ICOS^+^ ST2^+^ with gating based on FMO controls. *P < 0.05 (Two-way ANOVA). Data are representative of four independent experiments (means ± s.e.m).

Of note, neonatal mice displayed 3–4 fold more lung ILC2s (lineage^-^ CD45^+^ ICOS^+^ ST2^+^; gating strategy in S1 Fig; raw numbers in S3 Fig) at baseline (i.e. prior to infection) compared to adults (Fig 2a–2c). After infection, lung ILC2 percentages at 1 dpi were significantly higher in RSV-infected neonates compared to adults. Furthermore, neonatal ILC2s expressed more ST2 (Fig 2d). There was no change in the frequency of ILC2s at baseline or at 1dpi (Fig 2c) or in ST2 levels on those ILC2s at baseline or at 1dpi (Fig 2d) in adults.

### Modulation of IL-33 levels during primary RSV infection alters ILC2 numbers and IL-13 production at 1 dpi

**Fig 3:**
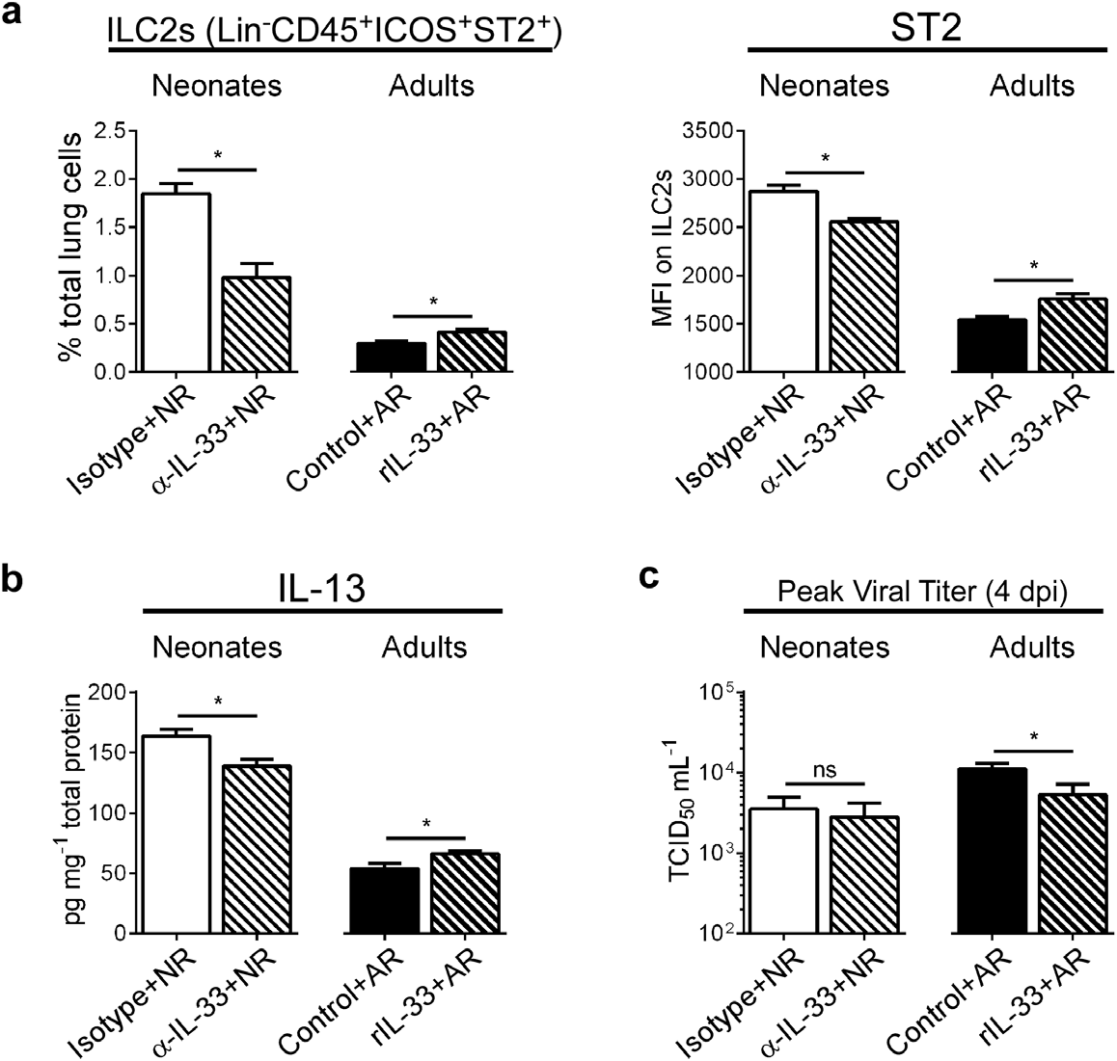
Modulation of IL-33 levels during primary RSV infection alters ILC2 numbers and IL-13 production at 1 dpi. (**a**) Number of ILC2s (lineage^-^ CD45^+^ ICOS^+^ ST2^+^) expressed as percentage of total lung cells and MFI of surface ST2 on ILC2s at 1 dpi in neonatal mice pretreated with IL-33 neutralizing antibody (α-IL-33 + NR) or control IgG antibody (Isotype + NR) and adult mice pretreated with recombinant IL-33 (rIL-33 + AR) or vehicle control (Control + AR). (*n* = 5–7 per group). (**b**) IL-13 protein levels in whole lung homogenates. (**c**) Pulmonary viral loads measured at 4 dpi (peak) using the TCID_50_ method. *P < 0.05 vs. indicated group, (Student’s *t*-test). Data are representative of two independent experiments (means ± s.e.m).

Because our data showed increased IL-33 in the lungs of RSV-infected neonates, but not adults, we pretreated neonates with anti-IL-33 antibody (α-IL-33+NR) and pretreated adults with recombinant IL-33 (rIL-33+AR) prior to RSV infection (see methods). When IL-33 was neutralized during primary RSV infection in neonates, the number of ILC2s and ST2 expression by those ILC2s was decreased compared to controls, while the opposite effect was seen in adults given rIL-33 (Fig 3a). Additionally, lung levels of IL-13 were statistically decreased/increased depending upon IL-33 neutralization/augmentation, respectively (Fig 3b). To determine if IL-33 modulation had an impact on overall viral load, we compared the amount of infectious virus in the lungs of treated and control mice at the peak of infection (i.e. 4 dpi) (Fig 3c). Interestingly, IL-33 neutralization had no effect on RSV burden in neonates, but we observed a significant decrease in viral load in adult mice pre-treated with rIL-33.

### IL-33 levels during primary RSV infection determine disease severity after reinfection

**Fig 4:**
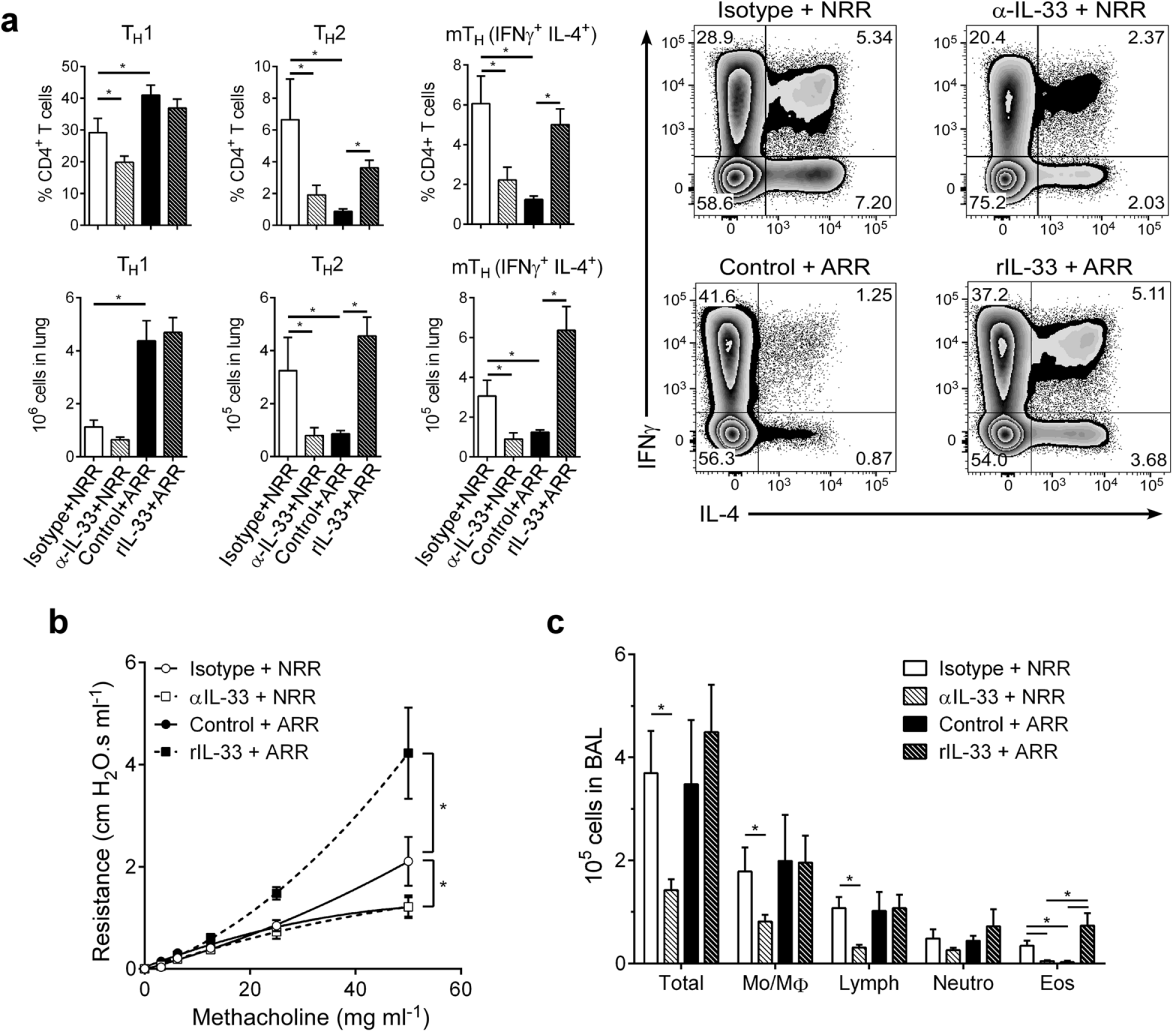
IL-33 levels during primary RSV infection determine disease severity after reinfection. (**a**) Number of Th1 (CD3^+^CD4^+^IFNγ^+^), Th2 (CD3^+^CD4^+^IL-4^+^), and multifunctional Th (CD3^+^CD4^+^IFNγ^+^IL-4^+^; mTh) cells in the lungs of mice at 6 dpi following reinfection with RSV (methods) (*n* = 5–6 per group); prior to initial RSV infection, neonatal mice were pretreated with IL-33 neutralizing antibody (α-IL-33 + NRR) or control IgG antibody (Isotype + NRR) and adult mice pretreated with recombinant IL-33 (rIL-33 + ARR) or vehicle control (Control + ARR). (**b**) Change in airway resistance in response to increasing doses of inhaled methacholine after treatment as in **a** (*n* = 6–8 per group). (**c**) Total cells (Total), monocytes/macrophages (Mo/MΦ), lymphocytes (Lymph), neutrophils (Neutro), and eosinophils (Eos) in BAL fluid after treatment as in **a** (*n* = 3–6 per group). *P < 0.05 vs. indicated group (one-way ANOVA with Bonferroni post-hoc tests; **a**, **c**) (two-way ANOVA with Bonferroni post-hoc tests; **b**). Data are representative of at least two independent experiments (means ± s.e.m).

In infants, severe RSV often causes bronchiolitis, recruitment of inflammatory cells (i.e., Th2 cells, eosinophils) to the lungs, and increased airway mucus, resulting in significant airway obstruction and airway hyperresponsiveness (AHR). A similar disease phenotype is readily observed (following reinfection) in mice initially infected with RSV as neonates, but not adults. To phenotypically recapitulate severe RSV infection seen clinically in human infants, mice from the same treatment groups as in Fig 3 were reinfected with RSV at 4 weeks post-primary infection (established neonatal mouse RSV infection + reinfection protocol [6, 7, 16]) and immune responses determined at 6 dpi (Fig 4). Neutralizing IL-33 during primary infection in neonates resulted in significant reductions in Th2 and multifunctional Th (mTh; IFNγ^+^, IL-4^+^) cells upon reinfection (α-IL-33+NRR) (Fig 4a). Conversely, administration of rIL-33 to adult mice during primary infection resulted in significant increases in Th2 and mTh cells upon reinfection (rIL-33+ARR), which were similar to levels induced in NRR mice.

Similar trends were observed in other markers of disease severity; when IL-33 was neutralized during primary RSV infection in neonates, development of AHR was abolished (Fig 4b). Interestingly, IL-33 administration to adult mice during primary infection resulted in exacerbated AHR, significantly above that of the NRR group. In the absence of RSV infection, neither IL-33 neutralization in neonates nor rIL-33 administration in adults resulted in AHR (S2 Fig). Cellularity in BAL from these same treatment groups displayed decreases in total cells, lymphocytes, and eosinophils in α-IL-33+NRR mice, and an increase in eosinophils in rIL-33+ARR mice (Fig 4c).

**Fig 5:**
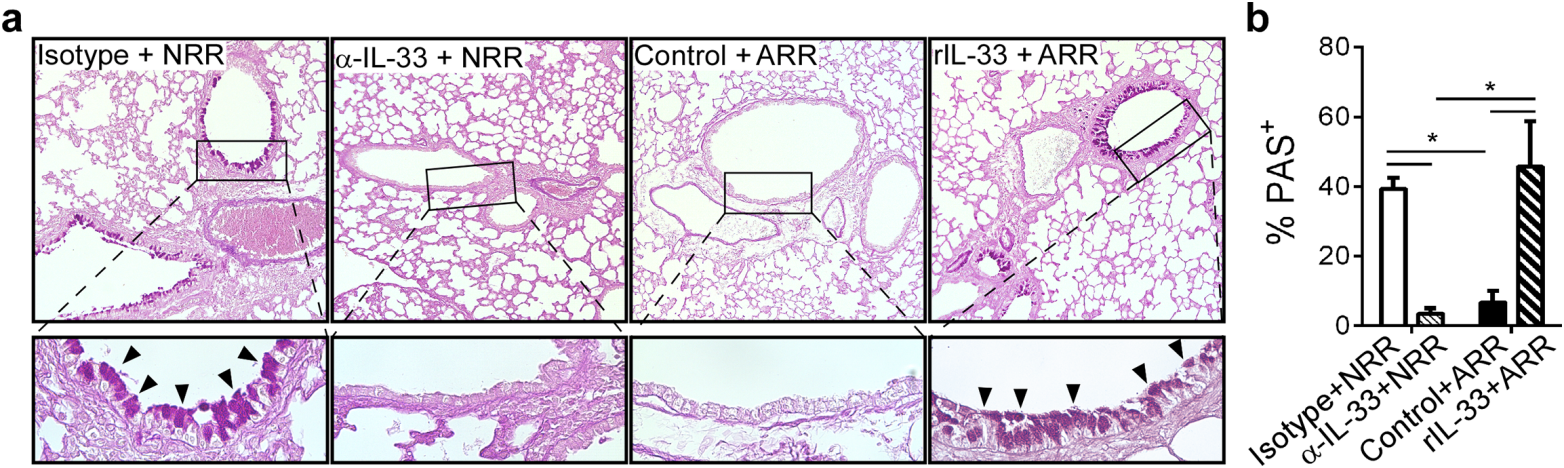
IL-33 levels during primary RSV infection determine disease severity after reinfection. (**a**) Lung sections obtained from mice treated as in Fig 4, stained with periodic acid-Schiff (PAS) to observe mucus (bright purple; indicated by black arrowheads). Upper images taken at 100X with inset (black box) magnified underneath to 400X. (**b**) Quantification of airway mucus in mice treated as in **a** (methods). *P < 0.05 vs. indicated group (one-way ANOVA with Bonferroni post-hoc tests). Data are representative of two independent experiments (means ± s.e.m).

Lung sections were stained with periodic acid-Schiff (PAS) to visualize mucus-producing cells in the airways. Airway mucus production was significantly decreased in α-IL-33+NRR mice compared to controls, while mucus was significantly increased in rIL-33+ARR mice compared to controls (Fig 5a and 5b).

### IL-33 signaling is required for neonatal RSV immunopathophysiology

**Fig 6:**
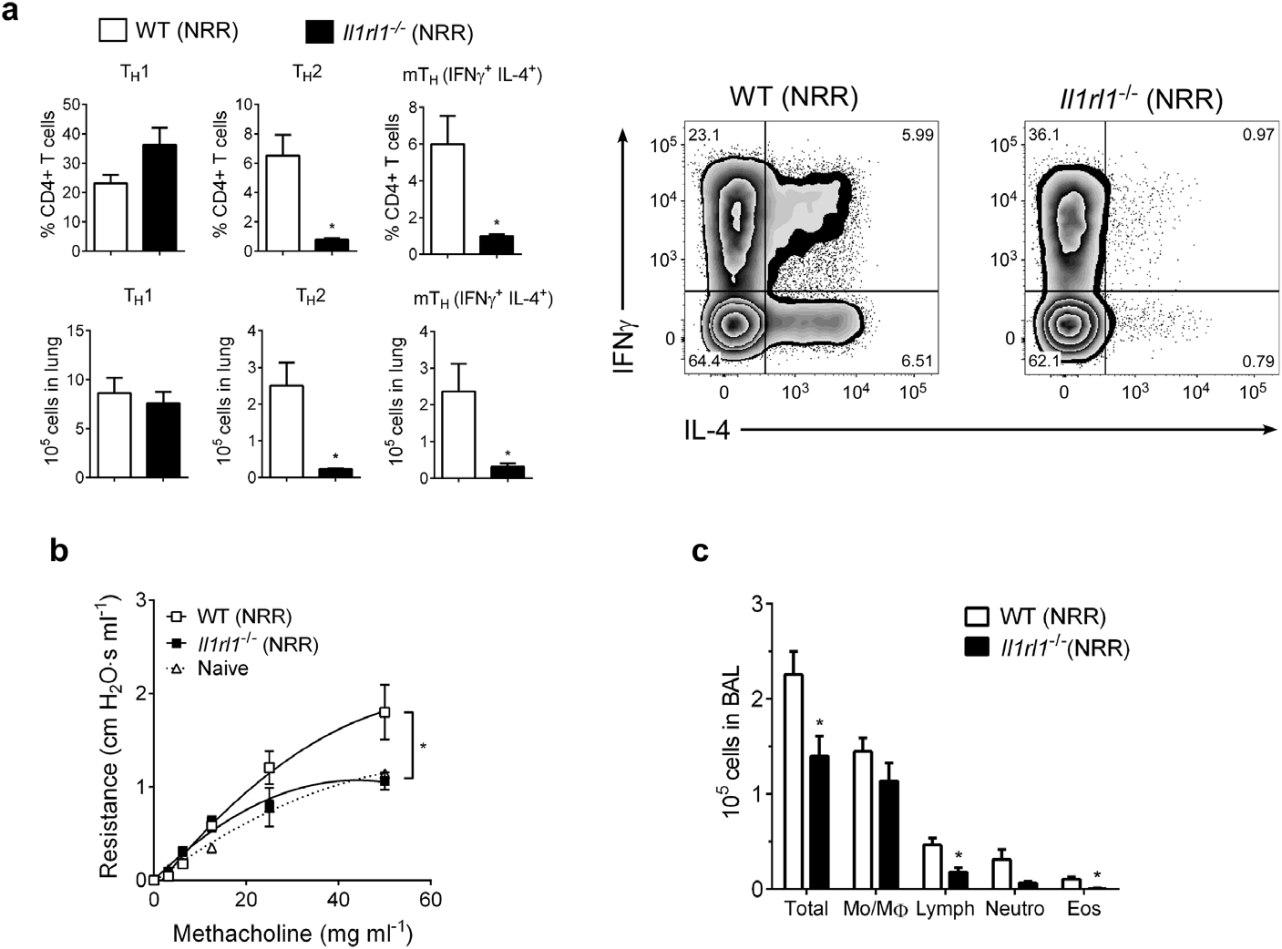
IL-33 signaling is required for neonatal RSV immunopathophysiology. (**a**) Number of Th1, Th2, and multifunctional mTh cells in the lungs of wild-type (WT) or ST2-deficient (*Il1rl1* ^-/-^) mice at 6 dpi following reinfection with RSV (methods) (*n* = 5–6 per group). (**b**) Change in airway resistance in response to increasing doses of inhaled methacholine after treatment as in **a** (*n* = 6 per group), compared to naïve control mice of similar size and age. (**c**) Total cells (Total), monocytes/macrophages (Mo/MΦ), lymphocytes (Lymph), neutrophils (Neutro), and eosinophils (Eos) in BAL fluid after treatment as in **a** (*n* = 5–8 per group). *P < 0.05 (Student’s *t*-test (**a**, **c**) or two-way ANOVA with Bonferroni post-hoc tests (**b**)). Data are representative of two independent experiments (means ± s.e.m).

To further demonstrate the role of IL-33 in promoting enhanced RSV disease in infected neonates, ST2 (IL-33 receptor, *Il1rl1* ^-/-^)-deficient neonatal mice were infected with RSV as in Figs 4, 5 and 6. Compared to similarly-infected wild type mice (WT NRR), *Il1rl1* ^-/-^ NRR mice failed to develop significant Th2 or mTh adaptive responses (Fig 6a), did not exhibit AHR (Fig 6b), and had decreased total cells, lymphocytes, and eosinophils in BAL (Fig 6c).

### IL-33 and IL-13 concentrations are elevated in nasal aspirates from infants hospitalized with RSV infection

**Fig 7:**
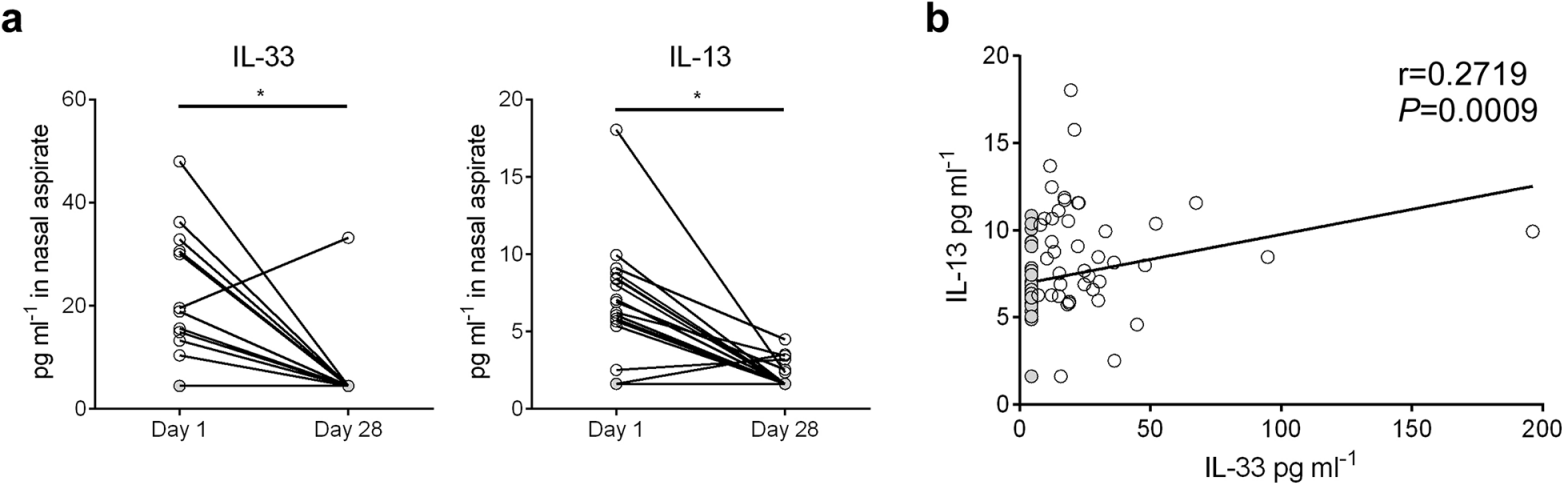
IL-33 and IL-13 concentrations are elevated in nasal aspirates from infants hospitalized with RSV infection. (**a**) Concentrations of IL-33 and IL-13 in nasal aspirates taken from RSV-infected infants (*n* = 19) on the first day (d1) of clinical presentation to Le Bonheur Children’s Hospital and again four weeks later (d28). (**b**) Correlation of IL-33 and IL-13 concentrations for all patients with d1 samples (*n* = 81). Samples with cytokine concentrations below the LOD of the assay are replaced by a value equal to the LOD divided by the square root of 2, and denoted with a filled circle. *P < 0.05 (Sign test (**a**) or Kendall’s tau correlation (**b**)).

Nasal aspirates were obtained from RSV-infected human infants (*n* = 81) on their initial day of clinical presentation (d1) at Le Bonheur Children’s Hospital. A follow-up nasal aspirate sample was obtained from some patients (*n* = 19) four weeks later (d28). Cytokine concentrations were analyzed in cell and mucus-free supernatants (Fig 7). In the paired samples, IL-33 and IL-13 levels were significantly elevated in d1 aspirates compared to d28 (Fig 7a). For all d1 samples, IL-33 levels significantly correlated with IL-13 (Fig 7b).

## Discussion

Infant susceptibility to severe RSV infection remains a significant global health burden for which no protective vaccine or adequate therapeutic is yet available. In recent years, it has become increasingly clear that age-dependent inherent differences in immunity play a vital role in both the magnitude [17, 18] and type [7] of responses to RSV. Interestingly, while infants are capable of mounting robust type-1 responses to other respiratory viruses such as influenza [19], they uniquely favor type-2 responses and the development of Th2 adaptive immunity (i.e. elevated IL-13 compared to IFN-γ) to RSV. Age at initial infection is critical in determining initial and subsequent immune responses to RSV [20] and in predicting subsequent wheeze/asthma [6, 7, 21, 22]. The role of age in dictating the response to RSV remains incompletely understood with respect to mechanism.

In the present study, we observed higher levels of lung ILC2s in neonates and an age dependent induction of ILC2s following RSV infection (i.e. ILC2s were only induced if the initial infection with RSV occurred in neonates). Our data resembles the seminal data demonstrating a role for ILC2s in the induction of AHR following influenza infection [15] with the exception that adult mice were infected with influenza and no data were presented on the Th subtypes following that infection or a subsequent infection. However, our data with RSV are in-line with recent data demonstrating an age dependent ILC2 response to rhinovirus in mice (i.e. increases in ILC2s in neonates but a minimal effect in adults) [23]. A potential difference between our results and those with neonatal rhinovirus is the cytokine responsible for driving ILC2 induction; with rhinovirus, ILC2s were IL-25 dependent whereas ILC2 induction with RSV appears to be IL-33 dependent and not IL-25 dependent. This is intriguing, although the mechanism at this time is unclear, since both RSV and rhinovirus cause severe infection and type-2 inflammation during infancy, and it is those with severe infection that are at greatest risk for long term airways disease [21]. Combined, the data from these studies expand the mechanistic knowledge of age-dependent type-2 immunity “bias” observed clinically in infants.

Severe RSV infection during infancy is heavily associated with the development of wheeze and/or allergic asthma during childhood and early adult life [21, 22, 24–26], and various genetic studies in humans have identified both IL-33 and ST2 as being key regulators in the development of asthma [11–14]. Our results demonstrate, for the first time, the vital age-dependent role of IL-33 in RSV-mediated Th2 immunopathophysiology. Levels of IL-33 during infection was influenced by age at the time of initial infection; and this, in turn, correlated with an increase in the numbers of lung ILC2s coinciding with enhanced IL-13 expression and increased lung dysfunction (i.e. AHR) and pathology. In this study, IL-33 neutralization during initial RSV infection in the neonate was sufficient to completely abolish the severe RSV phenotype (i.e., Th2 inflammation, AHR, eosinophilia, airway mucus), while administration of rIL-33 during initial infection in adult mice was sufficient to induce the severe RSV phenotype. Notably, these IL-33 effects during neonatal RSV infection appeared to be through changes in innate (i.e. ILC2) and adaptive (i.e. Th2) immunity and not via changes in viral load. The effects of rIL-33 during adult RSV also appeared to be mediated through innate and adaptive immunity; however, the counterintuitive decrease in viral load in rIL-33 treated mice suggests that its influence is more complex.

The strong Th2-promoting ability of IL-33-activated ILC2s has been largely demonstrated in animal models of Th2 disease such as asthma [10, 27] and helminth infection [8, 28]. It has been shown that ILC2s influence adaptive T cell responses both directly through MHCII-mediated crosstalk [29] and indirectly through Th2 cytokine production. Despite the severe immunopathophysiology we observed in rIL-33 treated adult mice infected with RSV, these mice displayed a significant yet relatively moderate increase in ILC2s during infection. Given the disparity in ILC2s between rIL-33 treated adult mice and neonatal mice, yet similar severity of RSV disease, it is probable that lung dendritic cells (DCs) are playing a large role in this model. In addition to activating ILC2s, IL-33 activates lung DCs via OX40L upregulation [30] to promote Th2 differentiation of naïve lymphocytes [31] and exacerbates eosinophilia [32]. Furthermore, blocking OX40L abrogates severe RSV in neonatal mice [33]. DCs are also important for anti-RSV responses, namely production of type-I interferons (i.e., IFNα, IFNβ), which are also impaired in RSV-infected infants [18, 34]. We have observed IL-13-mediated Th2 promotion by DCs in RSV (manuscript in preparation), and the rapidity of IL-13 expression following RSV infection suggests ILC2s as the source. We are currently investigating the ability of ILC2s, IL-33, and IL-13 to negatively influence type-I interferon production by pDCs. A role for DCs is currently being explored.

Though not as robust as earlier timepoints, a second, additional increase in IL-13 was observed at 6 dpi of initial infection. The significant increase in Th2 cells that has also been observed at 6 dpi suggests that they are at least partly responsible for elevated IL-13 levels at this time point.

The receptor for IL-33, ST2, also exists in a soluble secreted form (sST2), which scavenges “free” IL-33 [35]. One would expect that increased IL-33 scavenging ability would correlate with decreased disease severity, as mice that overexpress sST2 display resistance to IL-33-induced inflammation [36]. However, IL-33 has been shown to upregulate sST2 levels, which subsequently reduces IL-33 levels [37]. Interestingly, sST2 has been detected in higher concentrations in nasal aspirates of severely RSV-infected infants requiring mechanical ventilation compared to mild/moderately-infected, non-ventilated infants [13]. This same study also identified SNPs in the sST2-encoding gene in these individuals; one of which correlated with disease severity. While it is unclear if this SNP is responsible for inefficient binding/scavenging of IL-33 resulting in enhanced disease severity, it is also plausible that elevated sST2 levels reflect earlier high levels of IL-33 in infants. If the latter is true, then the timecourse of infection (i.e. days post-infection) at the time of sampling will be an important factor as future data is interpreted. In either case, these data cumulatively demonstrate a role for sST2 in RSV disease. The exact role of sST2 is our neonatal mouse RSV model was not tested and therefore it is difficult to reconcile this human data with our data demonstrating increased cell bound ST2 and IL-33 on ILC2s.

Surprisingly, early changes in RSV-induced IL-33 and ILC2s were age-dependent, as neonatal mice responded with robust IL-33 expression and a significant increase in ILC2s; while adult mice failed to induce IL-33 or ILC2s. Importantly, we observed elevated (and positively correlated) IL-33 and IL-13 in nasal aspirates of infants hospitalized with RSV infection. A limitation of our human data is the lack of samples from uninfected infants or infants infected with RSV but with mild (no medical intervention) or moderate (office visit) disease. With such samples, it is possible that these data would yield more robust conclusions (i.e. IL-33 correlates to RSV disease severity). The increase in IL-33 in nasal aspirates from infants along with our mouse data cumulatively suggest that IL-33 and ILC2s are critical for the age-dependent development of asthma following severe RSV infection during infancy and that they may be a plausible therapeutic target.

Infants that develop severe RSV bronchiolitis prior to 4 months of age are at increased risk to develop childhood asthma [38]. Differential regulation of either IL-33 or ILC2s as a factor of age is a previously unexplored area of developmental immunology that may further explain the Th2-biased immunity observed during early life. IL-33 has been reported to be constitutively produced (but not secreted), predominantly by non-hematopoietic (i.e., epithelial) cells in the lung [39, 40]. It is produced as a full-length form, which is biologically active but confined to the nucleus [41] where it functions as a transcriptional regulator [42]; this form can be released upon tissue damage or necrosis to act as an alarmin for the surrounding tissue [43] or be proteolytically cleaved into a highly active mature form by cathepsin G [44]. Furthermore, there is evidence that IL-33 is inactivated by caspase-1 [45, 46] which is increased during inflammasome activation [47]. Our data suggest that the airway epithelium is a significant source of IL-33 during neonatal RSV infection. Indeed, given the propensity for RSV to primarily infect the airway epithelium and the sheer number of epithelial cells in the lung, it is likely that the bulk of IL-33 secretion/release is epithelium-derived. Though outside the scope of the present study, we are investigating epithelial subsets within this context to determine which types of cells within the epithelium are responsible.

Another product of inflammasome activation, IL-1β, suppresses IL-33 and ILC2s during Th2-mediated disease [48]. Interestingly, we have previously demonstrated defective inflammasome activation in RSV-infected neonatal mice compared to adult mice and in human cord blood mononuclear cells (MNCs) compared to adult peripheral blood MNCs [49]. It is therefore possible that a lack of caspase-1 activity and/or IL-1β production results in “unchecked” IL-33 release in RSV-infected neonates/infants. Additionally, increased numbers of ILC2s have been observed in fetal human lungs compared to adult lungs [50] and in human cord blood MNCs compared to adult peripheral blood MNCs [51]. Despite these indications that IL-33 and ILC2s play a larger role in the immunity of neonates/infants, to date there have been no studies focused on when/how these factors change during early development and their subsequent influence on immune responses.

The present study defines roles for ILC2s and IL-33 in a previously unrecognized disease, and illuminates developmental variables as an area worthy of future consideration. The absence of IL-33 (or IL-33 signaling via knockout of its receptor) completely abolished the severe RSV disease phenotype in neonatal mice; while the administration of the cytokine during infection in adult mice resulted in exacerbated Th2 inflammation, lung dysfunction, and airway mucus. Age being a factor in IL-33 regulation has important implications for RSV research and pediatric immunology as a whole due to the involvement of IL-33 in many diseases affecting infancy and early childhood.

## Methods

### Mice

Mice were maintained in a specific-pathogen-free facility in ventilated microisolator cages. BALB/c mice were purchased from Harlan Laboratories (Indianapolis, IN). *Il1rl1* ^-/-^ mice [52] on a BALB/c background were generously provided by Dr. Hirohito Kita (Mayo Clinic) with approval from Dr. Andrew McKenzie (Medical Research Council, UK). Breeders were time-mated, and pups born on the same date were used for experiments. All animal protocols were prepared in accordance with the Guide for the Care and Use of Laboratory Animals and approved by the Institutional Animal Care and Use Committee at the University of Tennessee Health Science Center.

### RSV infection

RSV strain A2 (Advanced Biotechnologies; Columbia, MD) was propagated in Vero cells grown in serum-free media (HyClone; Thermo Fisher), harvested using standard protocol [16, 17], and stored at -80°C until use. Adult mice (6–8 week old) or neonatal mice (5 d old) were infected I.N. with RSV in serum-free media at a dose of 2 × 10^5^ 50% tissue culture infectious dose (TCID_50_) per gram of body weight. Reinfection with RSV occurred 4 weeks after the initial infection. Viral load was assessed in homogenized whole lung at 4 dpi by titer assay using the Spearman and Karber TCID_50_ method [53] with a lower detection limit of 316 TCID_50_ ml^-1^. For UV-RSV, virus from the same stock was inactivated by exposure to 1800 mJ UV light (Gene Linker; Bio-Rad; Hercules, CA). Inactivation was confirmed via viral titer assay.

### IL-33 treatments

Three hr prior to RSV infection, neonatal mice received IL-33 neutralizing antibody (α-IL-33; 2 mg kg^-1^ in PBS per supplier’s recommendation; I.N.). Controls received Rat IgG isotype control. Recombinant IL-33 (rIL-33; Peprotech) was administered to adult mice (0.5 μg in PBS 0.1% BSA) [27] I.N. at 24 hr and 3 hr before infection. The volumes of all neonatal and adult treatments were 10 μl and 50 μl, respectively. Controls received vehicle. IL-33 neutralizing antibody and isotype control were generously provided via MTA with Pfizer Inc.

### Cytokine expression

Previously frozen lung samples were homogenized in T-PER reagent (Thermo Fisher; Waltham, MA) in the presence of a protease inhibitor cocktail. Cytokine expression in whole lung homogenate was determined by Platinum ELISA (eBioscience; San Diego, CA) and corrected for total protein determined by BCA assay (Thermo Fisher).

### Flow cytometry

Lung single-cell suspensions [54] were stained with a fixable viability marker, stained with an antibody panel (below), and assayed on a FACS Canto II (BD; Franklin Lakes, NJ). For CD4 T cell population identification and determination of IL-33 cell sources, cells were stimulated for 5 hr with phorbol-12-myristate-13-acetate (PMA; 5 ng ml^-1^) and ionomycin (500 ng ml^-1^) in the presence of a protein transport inhibitor (GolgiPlug; BD) prior to intracellular staining with CD3—eFluor450, CD4—PerCP, IFNγ—PE, and IL-4—PE-Cy7 or CD45—PerCP-Cy5.5, EpCam—APC, CD31—PE-Cy7 (all eBioscience), and IL-33—PE (R&D Systems; Minneapolis, MN). For ILC2 staining, cells were labeled with antibodies to CD3—PacBlue, CD19—PacBlue, CD11c—PacBlue, CD11b—PacBlue, CD49b—PacBlue, F4/80—PacBlue, FcεRI—PacBlue, and Sca-1—PE-Cy7, (all BioLegend; San Diego, CA), ST2—FITC (MD Bioproducts; St. Paul, MN), Thy1.2 (CD90)—biotin (Southern Biotech; Birmingham, AL), Gata3—PE-Cy7 (BD), CD45—PerCP-Cy5.5, CD25—APC-Cy7/PE, and ICOS—APC (all eBioscience). Data were analyzed using FlowJo v10 (S1 Fig).

### Lung function test

In anesthetized (ketamine/xylazine, 180/10 mg kg^-1^), tracheotomized mice, peak airway resistance values in response to increasing doses of inhaled methacholine was measured using the flexiVent system (Scireq; Montreal, Canada).

### Bronchoalveolar cellularity

Mouse bronchoalveolar cells were isolated by lavage of the lungs with 1 ml PBS. Cells were then counted, spun onto slides, and stained with Hema-3 kit (Thermo Fisher). Differential cell counts were conducted in a blinded manner using standard morphological criteria.

### Lung histopathology and *in situ* hybridization

Mouse lungs were retroperfused with PBS, then gravity-inflated with zinc-formalin. Following fixation and paraffin embedding, 4 μm sections were prepared using standard procedures. Slides were stained with periodic acid-Schiff (PAS) to observe mucus production. As previously performed for quantification of mucus-producing cells [54], the number of PAS^+^ epithelial cells was divided by the total number of epithelial cells in a respective airway; this was repeated for at least 10 airways on each section, giving an average percentage. *In situ* hybridization of IL-33 mRNA was performed on deparaffinized sections with RNAscope technology (Advanced Cell Diagnostics; Hayward, CA) according to manufacturer’s instructions. All images were acquired using an EVOS FL Auto (Life Technologies; Grand Island, NY) microscope.

### Cytokine measurements in nasal aspirates

During the winter seasons of 2013–2015, previously healthy children (<2 yr of age) testing positive for RSV by antigen and/or PCR (and negative for influenza) at Le Bonheur Children’s Hospital were prospectively enrolled in a natural history study. Exclusion criteria included diagnosed immunodeficiency, chronic lung disease, congenital heart disease, or had received steroid treatment within the last month (patient demographics provided in S1 Table). Nasal aspirates were obtained on the first day of clinical presentation (median of 3 days after first symptom onset, IQR 2–5 days), and cell/mucus-free supernatants were stored at -80°C until analysis. A subset of patients had an additional nasal aspirate sample obtained four weeks later. Cytokine levels were determined by Milliplex human cytokine assay (Millipore, St. Charles, MO) according to manufacturer’s instructions. All returned values that were below the limit of detection (LOD) for the respective cytokine target were replaced with the LOD divided by the square root of 2 [55]. This study was conducted with approval of the Institutional Review Board of the University of Tennessee Health Science Center.

### Statistics

Data were analyzed with Prism5 (Graphpad; La Jolla, CA), and presented as means ± s.e.m. One-way or Two-way ANOVA with Bonferroni post-tests or student’s t-tests (both paired and unpaired) were used, where appropriate. *P* values <0.05 were considered significant. For nasal aspirate analyses, a Sign analysis was used for paired data and a Kendall’s tau correlation coefficient was computed to describe the correlation between IL-33 and IL-13.

### Ethics statement

All animal protocols were prepared in accordance with the Guide for the Care and Use of Laboratory Animals and approved (#14–045.0) by the Institutional Animal Care and Use Committee at the University of Tennessee Health Science Center.

## Supporting Information

S1 Fig ILC2 Gating Strategy with FMO controls. (JPG)

S2 Fig flexiVent analysis of αIL-33 and rIL-33 treated (non RSV-infected) controls. (PDF)

S3 Fig Raw ILC2 numbers from experiments shown in Figs 2a and 3b. (PDF)

S1 Table Characteristics of enrolled patient population. (PDF)

## References

[1] Openshaw PJ, Tregoning J, Harker J. RSV 2005: Global impact, changing concepts, and new challenges. Viral Immunol. 2005;18(4):749–51. Epub 2005/12/20. 10.1089/vim.2005.18.749 . 16359242

[2] Centers for Disease Control and Prevention. RSV | Trends and Surveillance | Respiratory Syncytial Virus | CDC http://www.cdc.gov/rsv/research/us-surveillance.html2014 [updated Dec 4, 2014; cited 2015 4/10/2015]. Available from: http://www.cdc.gov/rsv/research/us-surveillance.html.

[3] Johnson JE, Gonzales RA, Olson SJ, Wright PF, Graham BS. The histopathology of fatal untreated human respiratory syncytial virus infection. Mod Pathol. 2007;20(1):108–19. 10.1038/modpathol.3800725 . 17143259

[4] Thompson WW, Shay DK, Weintraub E, Brammer L, Cox N, Anderson LJ, et al Mortality associated with influenza and respiratory syncytial virus in the United States. JAMA. 2003;289(2):179–86. . 12517228 10.1001/jama.289.2.179

[5] Boyce TG, Mellen BG, Mitchel EF Jr., Wright PF, Griffin MR. Rates of hospitalization for respiratory syncytial virus infection among children in medicaid. J Pediatr. 2000;137(6):865–70. 10.1067/mpd.2000.110531 . 11113845

[6] Cormier SA, You D, Honnegowda S. The use of a neonatal mouse model to study respiratory syncytial virus infections. Expert review of anti-infective therapy. 2010;8(12):1371–80. 10.1586/eri.10.125 21133663 PMC3033119

[7] You D, Becnel D, Wang K, Ripple M, Daly M, Cormier SA. Exposure of neonates to respiratory syncytial virus is critical in determining subsequent airway response in adults. Respiratory research. 2006;7:107 10.1186/1465-9921-7-107 16893457 PMC1563465

[8] Neill DR, Wong SH, Bellosi A, Flynn RJ, Daly M, Langford TK, et al Nuocytes represent a new innate effector leukocyte that mediates type-2 immunity. Nature. 2010;464(7293):1367–70. 10.1038/nature08900 20200518 PMC2862165

[9] Halim TY, Krauss RH, Sun AC, Takei F. Lung natural helper cells are a critical source of Th2 cell-type cytokines in protease allergen-induced airway inflammation. Immunity. 2012;36(3):451–63. 10.1016/j.immuni.2011.12.020 . 22425247

[10] Barlow JL, Bellosi A, Hardman CS, Drynan LF, Wong SH, Cruickshank JP, et al Innate IL-13-producing nuocytes arise during allergic lung inflammation and contribute to airways hyperreactivity. The Journal of allergy and clinical immunology. 2012;129(1):191–8 e1–4. 10.1016/j.jaci.2011.09.041 . 22079492

[11] Tulah AS, Holloway JW, Sayers I. Defining the contribution of SNPs identified in asthma GWAS to clinical variables in asthmatic children. BMC medical genetics. 2013;14:100 10.1186/1471-2350-14-100 24066901 PMC3849932

[12] Akhabir L, Sandford A. Genetics of interleukin 1 receptor-like 1 in immune and inflammatory diseases. Current genomics. 2010;11(8):591–606. 10.2174/138920210793360907 21629437 PMC3078684

[13] Faber TE, Schuurhof A, Vonk A, Koppelman GH, Hennus MP, Kimpen JL, et al IL1RL1 gene variants and nasopharyngeal IL1RL-a levels are associated with severe RSV bronchiolitis: a multicenter cohort study. PloS one. 2012;7(5):e34364 Epub 2012/05/11. 10.1371/journal.pone.0034364 22574108 PMC3344820

[14] Bonnelykke K, Matheson MC, Pers TH, Granell R, Strachan DP, Alves AC, et al Meta-analysis of genome-wide association studies identifies ten loci influencing allergic sensitization. Nature genetics. 2013;45(8):902–6. 10.1038/ng.2694 . 23817571 PMC4922420

[15] Chang YJ, Kim HY, Albacker LA, Baumgarth N, McKenzie AN, Smith DE, et al Innate lymphoid cells mediate influenza-induced airway hyper-reactivity independently of adaptive immunity. Nat Immunol. 2011;12(7):631–8. 10.1038/ni.2045 21623379 PMC3417123

[16] Wang P, You D, Saravia J, Shen H, Cormier SA. Maternal exposure to combustion generated PM inhibits pulmonary Th1 maturation and concomitantly enhances postnatal asthma development in offspring. Particle and fibre toxicology. 2013;10(1):29. 23856009 10.1186/1743-8977-10-29 PMC3717277

[17] Cormier SA, Shrestha B, Saravia J, Lee GI, Shen L, DeVincenzo JP, et al Limited type I interferons and plasmacytoid dendritic cells during neonatal respiratory syncytial virus infection permit immunopathogenesis upon reinfection. J Virol. 2014;88(16):9350–60. 10.1128/JVI.00818-14 24920801 PMC4136292

[18] Marr N, Wang TI, Kam SH, Hu YS, Sharma AA, Lam A, et al Attenuation of respiratory syncytial virus-induced and RIG-I-dependent type I IFN responses in human neonates and very young children. J Immunol. 2014;192(3):948–57. 10.4049/jimmunol.1302007 . 24391215

[19] Naura AS, Hans CP, Zerfaoui M, You D, Cormier SA, Oumouna M, et al Post-allergen challenge inhibition of poly(ADP-ribose) polymerase harbors therapeutic potential for treatment of allergic airway inflammation. Clinical and experimental allergy: journal of the British Society for Allergy and Clinical Immunology. 2008;38(5):839–46. Epub 2008/02/12. 10.1111/j.1365-2222.2008.02943.x 18261157 PMC2740645

[20] DeVincenzo JP, Wilkinson T, Vaishnaw A, Cehelsky J, Meyers R, Nochur S, et al Viral load drives disease in humans experimentally infected with respiratory syncytial virus. Am J Respir Crit Care Med. 2010;182(10):1305–14. 10.1164/rccm.201002-0221OC 20622030 PMC3001267

[21] Sigurs N, Aljassim F, Kjellman B, Robinson PD, Sigurbergsson F, Bjarnason R, et al Asthma and allergy patterns over 18 years after severe RSV bronchiolitis in the first year of life. Thorax. 2010;65(12):1045–52. 10.1136/thx.2009.121582 . 20581410

[22] Carroll KN, Wu P, Gebretsadik T, Griffin MR, Dupont WD, Mitchel EF, et al Season of infant bronchiolitis and estimates of subsequent risk and burden of early childhood asthma. The Journal of allergy and clinical immunology. 2009;123(4):964–6. 10.1016/j.jaci.2008.12.011 19181372 PMC2707077

[23] Hong JY, Bentley JK, Chung Y, Lei J, Steenrod JM, Chen Q, et al Neonatal rhinovirus induces mucous metaplasia and airways hyperresponsiveness through IL-25 and type 2 innate lymphoid cells. The Journal of allergy and clinical immunology. 2014;134(2):429–39. Epub 2014/06/10. 10.1016/j.jaci.2014.04.020 24910174 PMC4119851

[24] Jackson DJ, Gangnon RE, Evans MD, Roberg KA, Anderson EL, Pappas TE, et al Wheezing rhinovirus illnesses in early life predict asthma development in high-risk children. Am J Respir Crit Care Med. 2008;178(7):667–72. 10.1164/rccm.200802-309OC 18565953 PMC2556448

[25] Stein RT, Sherrill D, Morgan WJ, Holberg CJ, Halonen M, Taussig LM, et al Respiratory syncytial virus in early life and risk of wheeze and allergy by age 13 years. Lancet. 1999;354(9178):541–5. 10.1016/S0140-6736(98)10321-5 . 10470697

[26] Sigurs N, Bjarnason R, Sigurbergsson F, Kjellman B, Bjorksten B. Asthma and immunoglobulin E antibodies after respiratory syncytial virus bronchiolitis: a prospective cohort study with matched controls. Pediatrics. 1995;95(4):500–5. . 7700748

[27] Barlow JL, Peel S, Fox J, Panova V, Hardman CS, Camelo A, et al IL-33 is more potent than IL-25 in provoking IL-13-producing nuocytes (type 2 innate lymphoid cells) and airway contraction. The Journal of allergy and clinical immunology. 2013;132(4):933–41. 10.1016/j.jaci.2013.05.012 . 23810766

[28] Halim TY, Steer CA, Matha L, Gold MJ, Martinez-Gonzalez I, McNagny KM, et al Group 2 innate lymphoid cells are critical for the initiation of adaptive T helper 2 cell-mediated allergic lung inflammation. Immunity. 2014;40(3):425–35. Epub 2014/03/13. 10.1016/j.immuni.2014.01.011 24613091 PMC4210641

[29] Oliphant Christopher J, Hwang You Y, Walker Jennifer A, Salimi M, Wong See H, Brewer James M, et al MHCII-Mediated Dialog between Group 2 Innate Lymphoid Cells and CD4+ T Cells Potentiates Type 2 Immunity and Promotes Parasitic Helminth Expulsion. Immunity. 2014 10.1016/j.immuni.2014.06.016 PMC4148706 25088770

[30] Murakami-Satsutani N, Ito T, Nakanishi T, Inagaki N, Tanaka A, Vien PT, et al IL-33 promotes the induction and maintenance of Th2 immune responses by enhancing the function of OX40 ligand. Allergol Int. 2014;63(3):443–55. 10.2332/allergolint.13-OA-0672 . 28942933

[31] Besnard AG, Togbe D, Guillou N, Erard F, Quesniaux V, Ryffel B. IL-33-activated dendritic cells are critical for allergic airway inflammation. Eur J Immunol. 2011;41(6):1675–86. 10.1002/eji.201041033 . 21469105

[32] Stolarski B, Kurowska-Stolarska M, Kewin P, Xu D, Liew FY. IL-33 exacerbates eosinophil-mediated airway inflammation. J Immunol. 2010;185(6):3472–80. 10.4049/jimmunol.1000730 . 20693421

[33] Han J, Dakhama A, Jia Y, Wang M, Zeng W, Takeda K, et al Responsiveness to respiratory syncytial virus in neonates is mediated through thymic stromal lymphopoietin and OX40 ligand. The Journal of allergy and clinical immunology. 2012;130(5):1175–86 e9 10.1016/j.jaci.2012.08.033 23036746 PMC3593657

[34] McIntosh K. Interferon in nasal secretions from infants with viral respiratory tract infections. J Pediatr. 1978;93(1):33–6. . 650342 10.1016/s0022-3476(78)80595-2

[35] Hayakawa H, Hayakawa M, Kume A, Tominaga S. Soluble ST2 blocks interleukin-33 signaling in allergic airway inflammation. The Journal of biological chemistry. 2007;282(36):26369–80. 10.1074/jbc.M704916200 . 17623648

[36] Ohto-Ozaki H, Kuroiwa K, Mato N, Matsuyama Y, Hayakawa M, Tamemoto H, et al Characterization of ST2 transgenic mice with resistance to IL-33. Eur J Immunol. 2010;40(9):2632–42. 10.1002/eji.200940291 . 20662097

[37] Ho JE, Chen WY, Chen MH, Larson MG, McCabe EL, Cheng S, et al Common genetic variation at the IL1RL1 locus regulates IL-33/ST2 signaling. J Clin Invest. 2013;123(10):4208–18. 10.1172/JCI67119 23999434 PMC3784527

[38] Wu P, Dupont WD, Griffin MR, Carroll KN, Mitchel EF, Gebretsadik T, et al Evidence of a causal role of winter virus infection during infancy in early childhood asthma. Am J Respir Crit Care Med. 2008;178(11):1123–9. 10.1164/rccm.200804-579OC 18776151 PMC2588491

[39] Liew FY, Pitman NI, McInnes IB. Disease-associated functions of IL-33: the new kid in the IL-1 family. Nature reviews Immunology. 2010;10(2):103–10. 10.1038/nri2692 . 20081870

[40] Smith DE. IL-33: a tissue derived cytokine pathway involved in allergic inflammation and asthma. Clinical and experimental allergy: journal of the British Society for Allergy and Clinical Immunology. 2010;40(2):200–8. 10.1111/j.1365-2222.2009.03384.x . 19906013

[41] Schmitz J, Owyang A, Oldham E, Song Y, Murphy E, McClanahan TK, et al IL-33, an interleukin-1-like cytokine that signals via the IL-1 receptor-related protein ST2 and induces T helper type 2-associated cytokines. Immunity. 2005;23(5):479–90. 10.1016/j.immuni.2005.09.015 . 16286016

[42] Carriere V, Roussel L, Ortega N, Lacorre DA, Americh L, Aguilar L, et al IL-33, the IL-1-like cytokine ligand for ST2 receptor, is a chromatin-associated nuclear factor in vivo. Proceedings of the National Academy of Sciences of the United States of America. 2007;104(1):282–7. 10.1073/pnas.0606854104 17185418 PMC1765450

[43] Lamkanfi M, Dixit VM. IL-33 raises alarm. Immunity. 2009;31(1):5–7. Epub 2009/07/17. 10.1016/j.immuni.2009.06.011 . 19604486

[44] Lefrancais E, Roga S, Gautier V, Gonzalez-de-Peredo A, Monsarrat B, Girard JP, et al IL-33 is processed into mature bioactive forms by neutrophil elastase and cathepsin G. Proceedings of the National Academy of Sciences of the United States of America. 2012;109(5):1673–8. Epub 2012/02/07. 10.1073/pnas.1115884109 22307629 PMC3277172

[45] Cayrol C, Girard JP. The IL-1-like cytokine IL-33 is inactivated after maturation by caspase-1. Proceedings of the National Academy of Sciences of the United States of America. 2009;106(22):9021–6. Epub 2009/05/15. 10.1073/pnas.0812690106 19439663 PMC2690027

[46] Luthi AU, Cullen SP, McNeela EA, Duriez PJ, Afonina IS, Sheridan C, et al Suppression of interleukin-33 bioactivity through proteolysis by apoptotic caspases. Immunity. 2009;31(1):84–98. Epub 2009/06/30. 10.1016/j.immuni.2009.05.007 . 19559631

[47] Franchi L, Eigenbrod T, Munoz-Planillo R, Nunez G. The inflammasome: a caspase-1-activation platform that regulates immune responses and disease pathogenesis. Nat Immunol. 2009;10(3):241–7. 10.1038/ni.1703 19221555 PMC2820724

[48] Zaiss MM, Maslowski KM, Mosconi I, Guenat N, Marsland BJ, Harris NL. IL-1beta suppresses innate IL-25 and IL-33 production and maintains helminth chronicity. PLoS pathogens. 2013;9(8):e1003531 Epub 2013/08/13. 10.1371/journal.ppat.1003531 23935505 PMC3731249

[49] Huang H, Saravia J, You D, Shaw AJ, Cormier SA. Impaired gamma delta T cell-derived IL-17A and inflammasome activation during early respiratory syncytial virus infection in infants. Immunology and cell biology. 2014 10.1038/icb.2014.79 . 25267484 PMC4323740

[50] Mjosberg JM, Trifari S, Crellin NK, Peters CP, van Drunen CM, Piet B, et al Human IL-25- and IL-33-responsive type 2 innate lymphoid cells are defined by expression of CRTH2 and CD161. Nat Immunol. 2011;12(11):1055–62. 10.1038/ni.2104 . 21909091

[51] Forsberg A, Bengtsson M, Eringfalt A, Ernerudh J, Mjosberg J, Jenmalm MC. GATA binding protein 3(+) group 2 innate lymphoid cells are present in cord blood and in higher proportions in male than in female neonates. The Journal of allergy and clinical immunology. 2014;134(1):228–30. 10.1016/j.jaci.2014.01.027 . 24636083

[52] Townsend MJ, Fallon PG, Matthews DJ, Jolin HE, McKenzie AN. T1/ST2-deficient mice demonstrate the importance of T1/ST2 in developing primary T helper cell type 2 responses. J Exp Med. 2000;191(6):1069–76. 10727469 10.1084/jem.191.6.1069 PMC2193113

[53] Kärber G. Beitrag zur kollektiven Behandlung pharmakologischer Reihenversuche. Archiv f experiment Pathol u Pharmakol. 1931;162(4):480–3. 10.1007/BF01863914

[54] Saravia J, You D, Thevenot P, Lee GI, Shrestha B, Lomnicki S, et al Early-life exposure to combustion-derived particulate matter causes pulmonary immunosuppression. Mucosal Immunol. 2013 10.1038/mi.2013.88 . 24172848 PMC3999175

[55] Croghan C, Egeghy PP, editors. Methods of Dealing With Values Below the Limit of Detection Using SAS. Southern SAS User Group; 2003; St. Petersburg, FL: US-EPA.

