# Supplementary figures and images for: Respiratory Syncytial Virus Disease Is Mediated by Age-Variable IL-33
Source: PLoS Pathog. 2015 Oct 16;11(10):e1005217. doi: 10.1371/journal.ppat.1005217 (PMC4608776; doi:10.1371/journal.ppat.1005217)

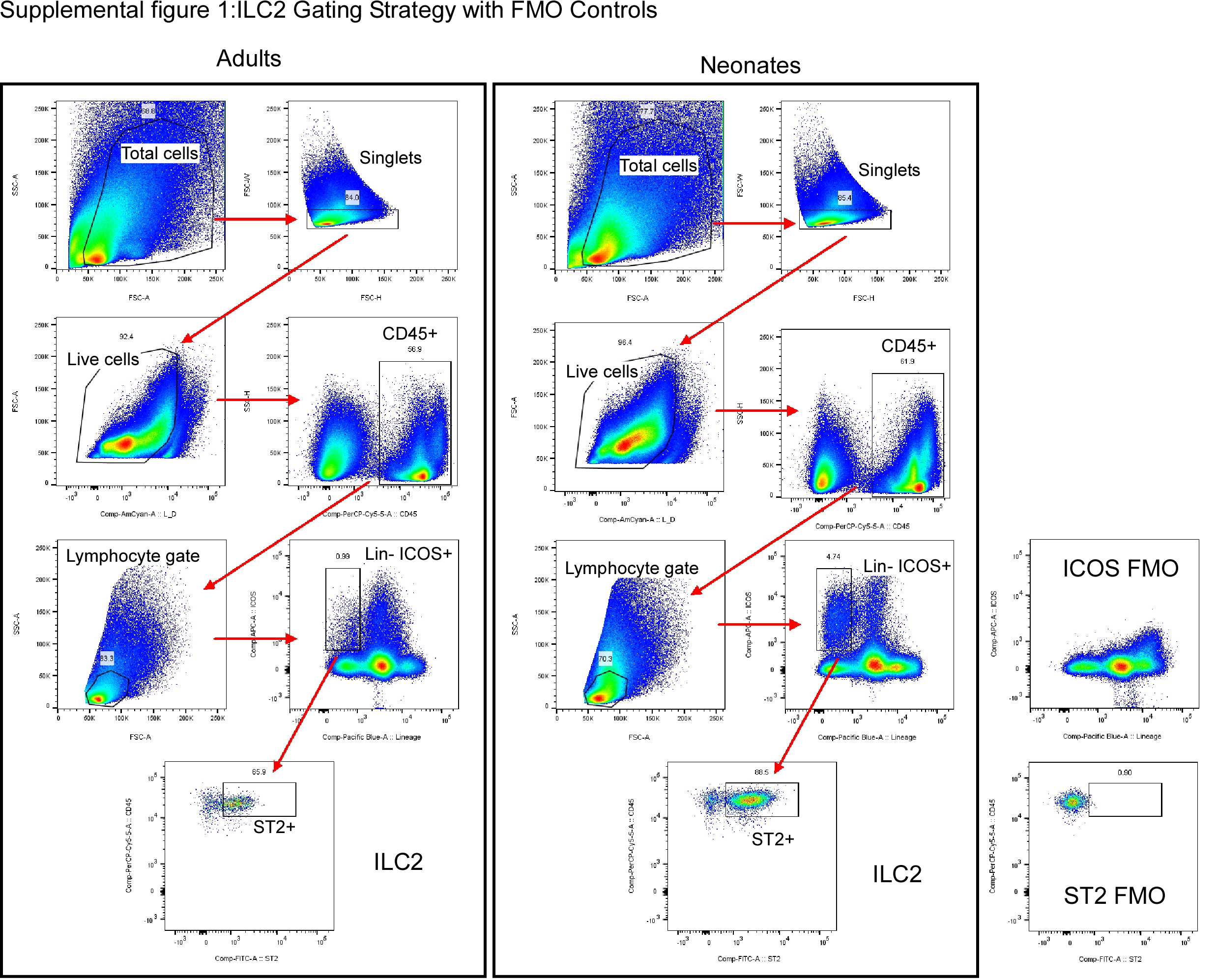

Supplement: S1 Fig — (JPG) [file ppat.1005217.s001.jpg]

Supplemental figure 2: flexiVent analysis of  $\alpha$ IL-33 and rIL-33 treated (non RSV-infected) controls

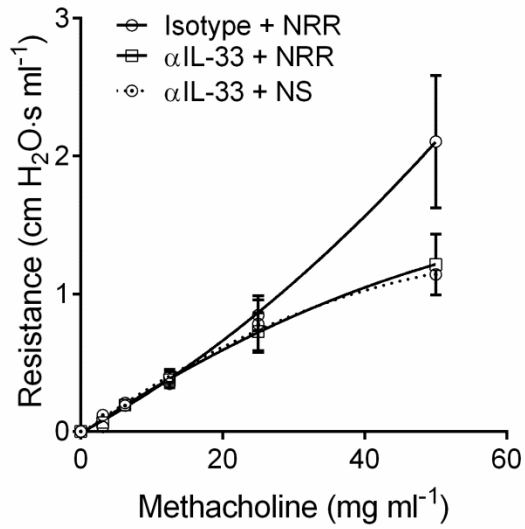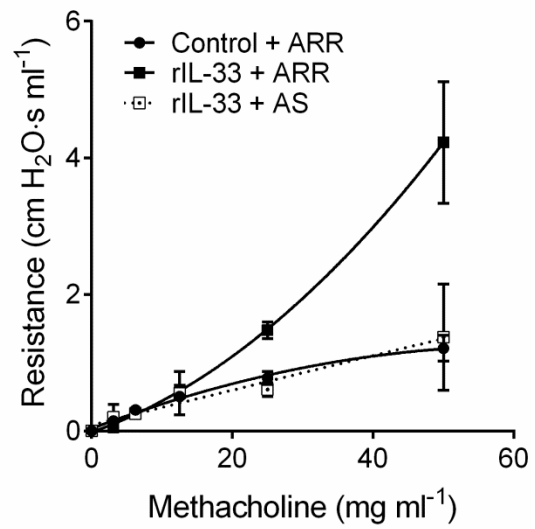

Supplement: S2 Fig — (PDF) [file ppat.1005217.s002.pdf]

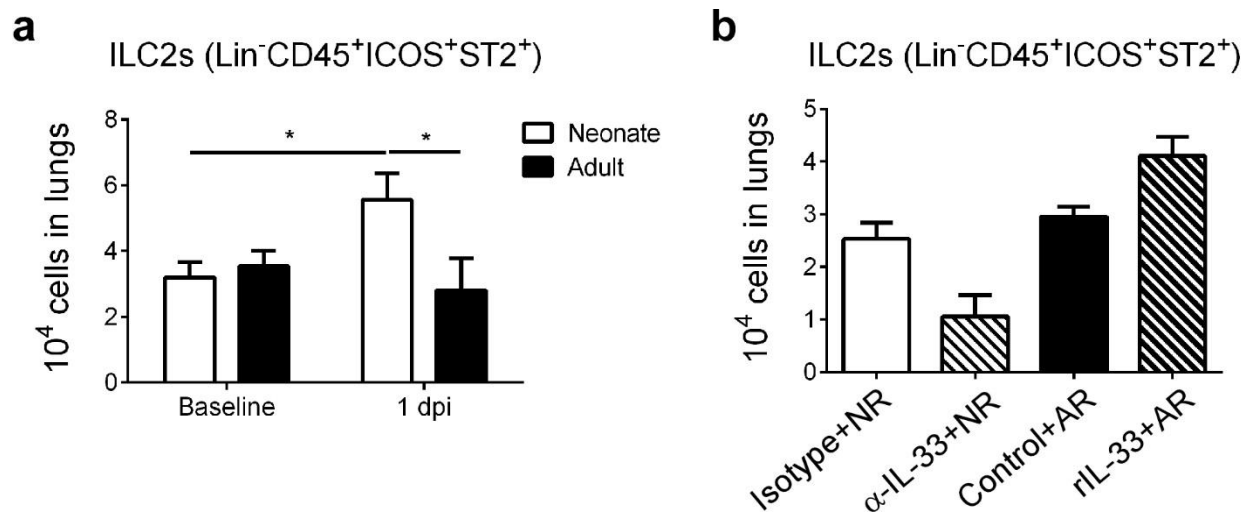

Supplemental figure 3: Raw ILC2 numbers from experiments shown in Fig 2 (a) and Fig 3 (b).

Supplement: S3 Fig — (PDF) [file ppat.1005217.s003.pdf]
