# Supplementary material for: Respiratory Syncytial Virus Disease Is Mediated by Age-Variable IL-33
Source: PLoS Pathog. 2015 Oct 16;11(10):e1005217. doi: 10.1371/journal.ppat.1005217 (PMC4608776; doi:10.1371/journal.ppat.1005217)
Supplement: S1 Table — (PDF) [file ppat.1005217.s004.pdf]

Supporting Information Table 1: Characteristics of Enrolled Patient Population

Infants	81
Age in days, median (range)	68 (9-686)
Male	48 (59)
Race/ethnicity	
White	23 (28)
Black	56 (69)
Hispanic	2 (3)
RSV test	
Antigen	78 (96)
PCR	3 (4)
Family history - Asthma	46 (57)
Family history - Atopy	6 (7)
Gestational age, Median (range), weeks	39 (32-41)
Birth weight, Median (range), grams	3104 (1106-6520)
Days after first symptom onset, median (IQR)	3 (2-5)

Note: Data are number (%) of total, unless otherwise indicated. RSV, respiratory syncytial virus; PCR, polymerase chain reaction; IQR, interquartile range.
